# Visual Attention Performances and Related Cerebral Microstructural Integrity Among Subjects With Subjective Cognitive Decline and Mild Cognitive Impairment

**DOI:** 10.3389/fnagi.2018.00268

**Published:** 2018-09-07

**Authors:** Min-Chien Tu, Chung-Ping Lo, Ching-Feng Huang, Wen-Hui Huang, Jie Fu Deng, Yen-Hsuan Hsu

**Affiliations:** ^1^Department of Neurology, Taichung Tzu Chi Hospital, Buddhist Tzu Chi Medical Foundation, Taichung, Taiwan; ^2^School of Medicine, Tzu Chi University, Hualien, Taiwan; ^3^Department of Radiology, Taichung Tzu Chi Hospital, Buddhist Tzu Chi Medical Foundation, Taichung, Taiwan; ^4^Department of Psychology, National Chung Cheng University, Chiayi, Taiwan; ^5^Center for Innovative Research on Aging Society (CIRAS), National Chung Cheng University, Chiayi, Taiwan

**Keywords:** diffusion tensor imaging, subjective cognitive decline, mild cognitive impairment, attention, cognition, continuous performance test

## Abstract

**Objective:** To compare visual attention performances and diffusion tensor imaging (DTI) between subjects with subjective cognitive decline (SCD) and mild cognitive impairment (MCI), and to discover neuronal substrates related to visual attention performances.

**Methods:** Thirty-nine subjects with SCD and 15 with MCI, diagnosed following neuropsychological tests and conventional brain magnetic resonance imaging, were recruited. All subjects were further examined by the Conners Continuous Performance Test 3 (CPT3) and DTI including fractional anisotropy (FA) and mean diffusivity (MD), in which group comparisons and stepwise linear regression were made.

**Results:** Subjects with MCI had a worse performance in all retrieval indices of verbal/nonverbal memory tests than those with SCD in the context of comparable general cognition and demographic status. In the CPT3, subjects with MCI had a significant longer hit reaction time (HRT) by univariate but not multivariate comparisons. Further analysis suggested that a longer HRT across all interstimuli intervals and at the point of fourth to sixth blocks were noted among MCI subjects. In DTI evaluations, FA value within the left forceps major was the only hotspot with significant between-group differences after the Bonferroni correction of FA and MD values. On the basis that HRT had significant inverse correlations with FA value within the genu of the corpus callosum and left forceps minor, regression analysis was conducted, showing HRT was best predicted by the FA value within the left forceps minor. Area under receiver operative characteristic curve was 0.70; the optimum cut-off for HRT was 515.8 ms, with a sensitivity of 85% but specificity of 47%.

**Conclusions:** Our report suggested that impaired sustained attention and vigilance to be an early cognitive marker in differentiating MCI from SCD, where MCI subjects had a longer HRT across all interstimuli intervals and more profoundly in later blocks. FA measures appeared to be more sensitive DTI parameters than MD values in detecting microstructural changes between SCD and MCI. The role of the anterior interhemispheric fibers in sustained attention implementation during visual signal detection task was highlighted.

## Introduction

Mild cognitive impairment (MCI) is a syndrome defined as cognitive decline beyond that anticipated from age-related changes (Gauthier et al., 2006), yet the affected individual does not meet the criteria for dementia (Albert et al., 2011). Current opinion has labeled MCI to be a risk state for dementia on the basis of 5%–15% annual dementia conversion rate (Mitchell and Shiri-Feshki, 2009). The concept of MCI underpins the importance in screening high risk groups by identifying factors that predict dementia onset within specific time periods. However, some people who are diagnosed having MCI may remain stable and even normalize in cognition over time (Gauthier et al., 2006), reflecting considerable heterogeneity of underlying pathogenesis under current diagnostic repertoire. In addition, the elderly with high education levels appear to be a group with high risk of being underdiagnosed. In their young adulthood, the cognitive performances of these individuals are often far above the average norm. In older age, they might report subjective cognitive decline (SCD) relative to their own baseline, while objectively still perform within the age-matched standards. Although subjects with SCD typically appear normal on standardized neuropsychological testing, this population has been gaining increasing attention as a pre-MCI condition based onto its close temporal relationship with dementia (Jessen et al., 2010). Of note, the risk of dementia conversion appeared to be more profound among those with worry or concerns (Jessen et al., 2014b). There are several lines of evidence supporting the unique role of SCD. In one epidemiology study, a high dementia conversion rate was concluded from longitudinal follow-up of a short period (2.6%–11.3% in 3 years; Jessen et al., 2010). In an autopsy report, there was a concrete relationship between memory complaints among non-demented elderly and Alzheimer’s disease (AD) pathology including amyloid plaques and neurofibrillary tangles (Barnes et al., 2006). Consistently, a longitudinal neuroimaging study has also identified independent association between SCD and subsequent hippocampal volume loss (Stewart et al., 2011). A clinician shall be aware that patients may present cognitive complaints at various timing points in the unfolding of dementia. Therefore, to better characterize either SCD or MCI through identifying potential markers is critically important, in hope that early intervention may slow down the progression of the disease (Sperling et al., 2011).

In cognition perspective, memory impairment has been proposed as one of the hallmarks in MCI, especially for those due to AD pathology (Albert et al., 2011). However, the cognitive deficits of non-memory domains also present, either in isolation or conjunction with memory impairment (McKhann et al., 2011). For example, attentional control deficits have been identified to progress among MCI and AD continuum, indicating impaired attention are highly likely to develop during the preclinical phase of AD (Belleville et al., 2007). Moreover, lowered attention among subjects with MCI not only led profound daily function disturbance (Klekociuk and Summers, 2014) but also was reported to be early indicators of possible transition to dementia (Saunders and Summers, 2011). Several researches have also implied that compromised attention is the early sign among subjects with SCD. For example, a novel neuroimaging study revealed compensatory functional activation changes during divided attention task among SCD individuals (Rodda et al., 2011). Subjects with SCD also appeared to have a P3 event-related potential different from the normal control in response to an attention control task (Smart et al., 2014). These findings indicated possible changes of neuronal substrates related to information processing among subjects with SCD. However, the kernel of the SCD diagnosis remained to be debatable, as some other researchers reported that depression and neuroticism accounted for considerable variance in cognitive complaints (Kliegel and Zimprich, 2005). In clinical practice, the individuals’ perceptions of cognitive problems are sometimes influenced by their affective status, which leads challenge in identifying the harbinger of ongoing neurodegeneration. Therefore, to identify a sensitive neuropsychological test in discerning MCI from SCD would further concretize the role of cognitive complaints, as the diagnosis of MCI carries a more imminent risk of dementia conversion.

In neuroimaging aspects, researchers have identified diffusion tensor image (DTI) to be an useful tool to discern microstructural changes between patients with MCI/AD continuum and the normal ageing (Teipel et al., 2011; Bosch et al., 2012; Tu et al., 2017). Due to the fact that DTI parameter changes might precede gray matter atrophy (Ibrahim et al., 2009; Selnes et al., 2012) and have significant correlation with cognitive performances (Grambaite et al., 2010), its application has been proven useful in predicting dementia conversion among MCI subjects (Haller et al., 2010). Furthermore, a novel study incorporating DTI and neurobiochemistry has identified a significant correlation between white matter microstructural changes and levels of cerebrospinal fluid biomarkers pathognomonic to AD (e.g., β-amyloid42 and total tau protein; Li et al., 2014). Studies recruiting subject with SCD further examined whether DTI has potential value to be an early marker for pre-MCI stage. However, the results have been inconsistent. Some DTI studies identified widespread white matter microstructural changes in SCD subjects evidenced by certain DTI parameters modification (Selnes et al., 2012). Others concluded that although SCD subjects displayed a trend of intermediate stage between normal ageing and MCI, the structural network during SCD stage was relatively preserved (Wang X. N. et al., 2016). Interestingly, still some other study revealed no significant changes of white matter integrity between normal ageing and SCD (Kiuchi et al., 2014). Regarding DTI clinical relevancy, there has been valuable information in delineating visual attention network in the context of lesion-based study. Two cohorts with post-stroke neglect indicated that microstructural derangement within contra-lesional frontoparietal connections (e.g., inferior/superior parietal lobe and left corpus callosum/cingulum) and bilateral occipital connections were important for visual attention system processing (Umarova et al., 2014, 2017). Some other research also identified dissociable circuits, mostly confined within the superior longitudinal fasciculus, inferior fronto-occipital fasciculus, and corpus callosum, responsible for post-stroke neglect (Vaessen et al., 2016). In a study incorporating DTI with positron emission tomography, associations between microstructural changes within the superior longitudinal fasciculus and metabolic changes within the inferior parietal and frontal eye field regions were regarded to be responsible for visuospatial dysfunction among subjects with posterior cortical atrophy (Cerami et al., 2015). Research focusing associations between DTI modifications and attention performances among subjects with MCI/SCD, however, is limited.

Although the importance of comprehensive assessment in both memory and non-memory functions in categorizing MCI and SCD has been widely advocated, controversy remains due to the fact that study design using same batteries might create a discussion obsessed by circular reasoning. If individuals with cognitive complaints were classified according to specific neuropsychological tests, then it would be less surprising that cross-sectional analysis of their profiles reveals lowered performances within the same domains. Likewise, differentiation subjects with SCD from those with MCI based onto neuropsychological tests would also affect the results of neuroimaging comparisons. To deploy a sensitive neuroimaging tool and neuropsychological tests distinct from those primarily used for classification may further clarify the differences of cognitive profiles between SCD and MCI. Additionally, cerebral microstructural changes expected to be associated with AD pathology could be further clarified through neuroimaging and neuropsychological characterization. Therefore, our study was aimed to: (i) compare visual attention test performances and whole-brain DTI profiles between subjects with SCD and MCI diagnosed by standardized neuropsychological tests and (ii) examine the clinical relevancy between visual attention performances and DTI parameters.

## Materials and Methods

### Participants

Thirty-nine subjects with SCD and 15 with MCI were enrolled from the Neurology outpatient clinic of our hospital. Data on demographics, serology tests, general cognitive function assessments, the CPT, and brain MRI (including DTI) studies were recorded for each subjects. This study was approved and carried out in accordance with the recommendations of Research Ethics Committee of the Taichung Tzu Chi Hospital, Buddhist Tzu Chi Medical Foundation (REC 104-05), with written informed consent from all subjects. All subjects gave written informed consent in accordance with the Declaration of Helsinki.

### Inclusion and Exclusion Criteria

All subjects were of age ≥50 years-old and formal education ≥6 years. The criteria specific for MCI were organized based onto the conservative criteria proposed by Jak et al. (2009) in order to maintain diagnostic stability, including: (i) subjective memory complaints yet preserved autonomy in activities of daily living; (ii) normal general cognitive function, as evidenced by the Taiwanese Mini-Mental State Examination (MMSE) ≥26 (Shyu and Yip, 2001); and (iii) at least two test performances within a cognitive domain falling 1.5 *SD* below the average level with respect to age- and education-adjusted normative data. The inclusion criteria of SCD followed (i) and (ii), but not (iii). In other words, subjects with SCD should have less cognitive deficits and they did not fulfill criteria (iii) for MCI. The exclusion criteria were: (1) not demented (McKhann et al., 2011); (2) Hachinski Ischemic Score >4 (Hachinski et al., 1975); (3) known history of psychiatric or other neurological illness (e.g., major depressive disorder, substance use, traumatic brain injury, neurodegenerative disease, or delirium); (4) severe loss of vision, hearing, or communicative ability; (5) intolerability on taking neuropsychiatric assessment and MRI examination, and known; (6) derangements in serology tests that may have contributed to cognitive impairment such as abnormal levels of free T4, cortisol, folic acid, vitamin B12, or rapid plasma reagin; (7) remarkable white matter T2-hyperintensities on MRI, defined as the Fazekas scale >2 (Fazekas et al., 1987). All subjects were free from psychotropic agents, and were required to refrain from the use of alcohol and drinks containing caffeine for at least 12 h before neuropsychological and visual attention tests.

### Demographic Data Registry

In addition to basic demographic data, the cognitive symptoms were detailed according to framework proposed by Jessen et al. (2014a). Briefly, this structural evaluation mainly focused onto categorizing cognitive symptom (e.g., frequent missing an appointment, forgetfulness, poor efficiency on work, etc.), the severity and duration of targeted symptoms, associated daily function impairment and confirmatory collateral information, if any. In addition, the Functional Activities Questionnaire was measured in order to rate these subjects’ abilities. Cut-point of nine in sum scores indicates impaired function, and has been applied for distinguishing demented individuals from normal aging (Pfeffer et al., 1982).

### Serology Test

Antecubital venous blood samples were collected after an 8-h fast for hemogram, serum creatinine, folate, vitamin B12, free T4, thyroid stimulating hormones, cortisol, and rapid plasma reagin measurements. Samples were collected in evacuated tubes containing EDTA, centrifuged within 10 min and stored below −20°C until analysis.

### Neuropsychological Tests

Neuropsychological tests were administrated by licensed clinical psychologists. In general cognitive function, the Taiwanese MMSE (Shyu and Yip, 2001), Cognitive Abilities Screening Instrument (CASI; Lin et al., 2012) and CDR- sum of box (Morris, 1993) were scored. According to the comprehensive criteria for MCI diagnosis (Jak et al., 2009), at least two test indices were used for judging a deficit in any given cognitive domain. Memory function was assessed by three indices of the Chinese version of the Verbal Learning Test (Chang et al., 2010), including the total score of immediate recall, the 10-min delay free recall, and the recognition score. Executive function was assessed by the Trail Making Test part B (Reitan, 1958), the backward span of the Digit Span subtest of the Chinese version of the Wechsler Adult Intelligence Scale (WAIS)— the 3rd edition (Chen and Chen, 2002), and two indices of the modified Cart Sorting Test (Nelson, 1976), including the number of category completed and the number of perseveration. Attention function was assessed by the Trail Making Test part A (Reitan, 1958), the forward span of the Digit Span (Chen and Chen, 2002), and the Digit Symbol Coding subtest of the WAIS (Chen and Chen, 2002). Language function was evaluated by the Neurosensory Center Comprehensive Examination for Aphasia (NCCEA) Object Naming (Hua et al., 1997), as well as the 30-s animal fluency task and the Language indices of the CASI (Lin et al., 2012). Additional tests were also given to gather more information of participants’ cognitive status, including the Visual Reproduction subtest of the Chinese version of the Wechsler Memory Scale (Hua et al., 2005), the Taiwan version of the Frontal Assessment Battery (FAB; Wang T. L. et al., 2016), and the Judgment of Line Orientation (Benton et al., 1994). The Beck Anxiety Inventory (Julian, 2011) and Beck Depression Inventory (Beck et al., 1988) were assessed for the severity of anxiety and depression symptoms of our subjects. Higher total scores in both inventories indicate more severe symptoms.

### Visual Attention Tests

The Conners Continuous Performance Test 3 (CPT 3; Conners, 2002) was administered in a quiet testing room. The test takes around 15 to 20 min, and subjects are asked to click a mouse button in response to seeing target stimuli that appear on a screen as soon as possible anytime when any letter appears with the exception of letter X. When X appears, the examinee is instructed to refrain from responding to non-target stimulus. The interstimuli intervals (ISIs) are 1 s, 2 s, 4 s with a display time of 250 ms. The test includes six different blocks, with three sub-block each consisting of 20 trials. Several following raw scores are derived after subject completed the entire administration. Generally, higher raw scores indicate worse performance.

The test encompasses the following parameters:

(i) Detectability: a measure representative the ability to discriminate non-targets (i.e., the letter X) from targets (i.e., all other letters).
(ii) Omissions: missed targets.
(iii) Commissions: incorrect responses to non-targets.
(iv) Perseverations: responses that are made in less than 100 ms following the presentation of stimulus.
(v) Hit reaction time (HRT): the response speed measured in milliseconds. Longer HRT indicates slowing of response speed. HRT serves to be one of the measures of both sustained attention and vigilance.
(vi) Hit reaction time standard deviation (HRTSD): the consistency of response speed to targets for the entire administration.
(vii) Variability: the response speed consistency within respondent.

Error measurements (i.e., omissions, commissions and perseverations) were presented with a percentage value to all stimuli. HRT and HRTSD were also analyzed according to each individual block change (BC) and ISI changes (e.g., HRT_BC, HRT_ISI), in order to explicitly assess sustained attention and vigilance, respectively.

### Brain Magnetic Resonance Imaging (MRI) and Diffusion Tensor Imaging (DTI)

All patients received brain MRI using a 3.0 T scanner (Discovery MR750, GE Medical System, Milwaukee, WI, USA) with an 8-channel phased array head coil in accordance with same imaging protocol, including axial T1-weighted imaging, T2 fluid-attenuated inversion recovery (T2-FLAIR), diffusion weighted imaging, and MR angiography for the circle of Willis. DTI data were acquired using a single-shot spin-echo echo-planar imaging sequence. The diffusion-sensitizing gradients were applied along 20 non-collinear directions with diffusion weighting factor *b* = 1,000 s/mm^2^, plus one *b* = 0 image. Parallel imaging technique Array Spatial Sensitivity Encoding Technique (ASSET) was utilized for reducing susceptibility to geometric artifacts and the option of dual spin-echo was applied to alleviate the eddy-current induced distortion. The remaining imaging parameters were: TR/TE = 8,000/82 ms, matrix size = 128 × 128, field of view (FOV) = 240 mm, slice thickness = 3 mm with no intersection gap, number of excitations = 2, number of slices = 67, scan time = 5 min and 58 s. All of the DTI images were sent to a workstation Advantage Windows 4.4 and processed using the FuncTool software (GE Healthcare, Milwaukee, WI, USA). Before DTI parametric maps of fractional anisotropy (FA) and mean diffusivity (MD) were generated, all diffusion data were with the affine and rigid body registrations to the image (*b* = 0) for reducing the motion and distortion.

FA and MD values were measured from 19 circular priori-defined subcortical regions of interest (ROI) as the template defined by Tu et al. (2017). The ROI size was kept consistent (30–35 mm^2^) in all of the subjects in order to obtain a stable number of voxels and decrease variance in the DTI parameters. In brief, midline ROIs included the genu, body (three portions) and splenium of the corpus callosum. Other ROIs including the superior longitudinal fasciculus, forceps minor, forceps major, anterior thalamic radiation, uncinate fasciculus, inferior longitudinal fasciculus, and cingulum, were evaluated symmetrically within bilateral hemispheres. The ROIs were manually drawn by a single rater (Min-Chien Tu), and the DTI parameters of each white matter tract were obtained from the averaged ROIs of two adjacent slices.

### Statistical Analysis

Comparisons between these two groups were made. The Shapiro-Wilk test was used to test normality of each variable. The independent *T*-test was used for those who were normally distributed, while the Mann Whitney *U* tests were used for those examined to be nonparametric variables. The *χ*^2^ test was used to detect group differences in demographic data. Cohen’s *d* was used to determine the effect size on comparing two groups of similar size and standard deviation. A Cohen’s *d* value of 0.20, 0.50 and 0.80 corresponds to the effect that could be described as small, median, and large, respectively (Levene, 1960). To determine the reliability of DTI measurements, the same rater repeated ROI selections on all subjects in this study. The intra-observer reliability was assessed using the averages of intra-class correlation coefficients (ICCs) with absolute agreement. In current study, we used a “Two-Way Random” effects model with absolute agreement. This model fits the condition that the dependent variables (DTI parameters) are assessed by the same rater, where both an effect of rater and of ratee (i.e., two effects) is considered and both rater and ratee are drawn randomly from larger populations (i.e., a random effects model). The ICCs values were considered to indicate excellent agreement and substantial agreement if they were greater than 0.8 and 0.60–0.79, respectively (Shrout and Fleiss, 1979). Three types of variance were derived from the ICCs analysis: Var (β) is variability due to differences in the subjects; Var (ε) is variability due to differences in the evaluations of the subjects by the judges; Var (α) is variability due to differences in the rating scale used by the judges. Pearson correlation was applied to determine the association between visual attention tests and DTI parameters; partial correlation analysis was used to further examine the results with the aim of controlling for confounding factors including age and education (Conners, 2002). In addition, to examine the effect of microstructural changes within ROIs on the performances of visual attention tests, stepwise linear regressions between targeted attention function and DTI parameters of all ROIs were tested. Receiver operating characteristic (ROC) curve was created to test the clinical utility of target CPT3 parameter, and to set the cut-off score. Optimum cut-off score for the target CPT3 parameter was determined at the maximum Youden index (J = sensitivity + specificity − 1) level (Akobeng, 2007). The area under the curves (AUC) was examined to show sensitivity and specificity of the target CPT3 parameter for identifying MCI. It is generally accepted that an AUC value greater than 0.9 indicates high accuracy, 0.7–0.9 indicates moderate accuracy, and 0.5–0.6 indicates low accuracy (Akobeng, 2007). All statistical tests were performed using SPSS software version 19 (IBM, Armonk, NY, USA). A *p* value less than 0.05 was considered to be statistically significant. The Bonferroni correction would be applied for statistical tests with multiple comparisons problems (Armstrong, 2014).

## Results

Table 1 shows basic information of subjects with SCD and MCI. Both groups showed comparable demographic profiles including age, age of onset, symptom duration, education, and gender (*p* = 0.183–0.716). Regarding descriptions of cognitive symptoms, most subjects reported remarkable concern (*n* = 47; 87%), and their medical help seeking behavior had associations with their cognitive problems (*n* = 54; 100%). A considerable portion of subjects (*n* = 45; 83%) reported a feeling that their performance to be worse than those of similar ages. Overall, there were eighty-nine and eighty percent of subjects who reported memory (*n* = 48) and non-memory (*n* = 43) complaints, respectively. Executive complaints represented the majority of non-memory complaints, accounting for forty-nine and forty-seven percent among subjects with SCD and MCI, respectively. The Functional Activities Questionnaire in both groups ranged from 0 to 7, and their average scores in subjects with SCD and MCI were 1.1 and 1.6, respectively. There was no differences of scores in the Beck Anxiety Inventory and Beck Depression Inventory (*p* = 0.857–0.945). Regarding performances of cognitive tests, both groups had a comparable general cognition status (*p* = 0.832–0.876). Ten, three and two subjects were categorized as single-domain amnestic, multi-domain amnestic and non-amnestic MCI, respectively. Among tests specific for individual cognitive domains, only the CVVLT and Wechsler Memory Scale showed significant between-group differences, in which subjects with MCI had worse performance in all subsets of recall than those with SCD (*p* = 0.043~ <0.001).

**Table 1:** Basic information of patients with SCD and MCI.

	SCD (*n* = 39)	MCI (*n* = 15)	*P* value
**Demographic data**
Age (years–old)	62.7 (7.61)	61.7 (7.75)	0.666
Age of onset (years–old)	61.5 (7.77)	60.7 (7.52)	0.716
Duration (years)	1.2 (1.39)	1.1 (0.94)	0.691
Education (years)	10.3 (3.10)	9.0 (3.40)	0.183
Gender (Male/Female)	14/25	7/8	0.467
Handedness (Right/Left)	38/1	15/0	-
**Cognitive symptoms (*n*)**			
Association with medical help seeking	39	15	-
Memory	35	13	0.747
Nonmemory	31	12	0.966
Execution	19	7	0.892
Attention	13	2	0.141
Visuospatial construct	4	3	0.339
Language	13	4	0.636
Concerns	33	14	0.392
Feeling worse than others	34	11	0.221
Impairment of daily function	36	14	0.897
Functional Activities Questionnaire	1.1 (1.8)	1.6 (2.1)	0.480
**Neuropsychological tests**			
Mini-Mental State Examination	28.2 (1.17)	28.2 (1.58)	0.876
Cognitive Abilities Screening Instrument	88.5 (13.80)	87.8 (6.16)	0.832
Clinical Dementia Rating—sum of box	0.7 (0.75)	1.0 (0.88)	0.188
Chinese Version Verbal Learning Test—total immediate recall	27.5 (3.44)	23.5 (2.82)	**<*0.001***
—recall after 30 s	7.5 (1.56)	6.4 (1.40)	***0.015***
—recall after 10 min	7.2 (1.11)	5.4 (1.50)	**<*0.001***
Visual Reproduction subtest of the —immediate recall	72.7 (13.75)	59.4 (21.87)	***0.010***
Wechsler Memory Scale —delayed recall	47.6 (19.68)	34.6 (22.55)	***0.043***
Frontal Assessment Battery	13.8 (1.74)	13.3 (2.32)	0.389
Object naming	15.7 (0.53)	15.3 (0.97)	0.109
Digit span	19.4 (4.03)	16.9 (5.49)	0.066
Judgement of Line Orientation	21.2 (4.25)	18.9 (7.02)	0.158
Beck Anxiety Inventory	11.7 (9.69)	11.6 (7.71)	0.945
Beck Depression Inventory	11.1 (8.72)	11.6 (7.13)	0.857

*MCI, mild cognitive impairment; SCD, subjective cognitive decline. Data presented as Mean (Standard deviation) unless stated elsewhere. No significant differences were noted by using the independent T-test and Chi-square test where appropriate. P values < 0.05 are in bold and italicized*.

Table 2 shows comparisons of CPT3 tests. Of note, subjects with MCI had a significant longer HRT (*p* = 0.013; Cohen’s *d* = 0.76) and wider HRTSD (*p* = 0.010; Cohen’s *d* = 0.70) than those with SCD by univariate comparisons. No significant between-group differences were noted after the Bonferroni corrections, with a threshold selected to be 0.05/9 = 0.0055. There was a positive correlation between HRT and HRTSD (ρ = 0.651; *p* < 0.001), indicating HRTSD turned to be wider in line with longer HRT.

**Table 2:** Comparisons of the continuous performance test (CPT) between patients with SCD and MCI.

	SCD (*n* = 39)	MCI (*n* = 15)	*P* value
**CPT raw scores**			
Detectability	−3.1 (0.77)	−3.0 (1.00)	0.717
^†^Omissions (%)	1.9 (3.72)	2.7 (4.80)	0.526
^†^Commissions (%)	21.2 (15.33)	22.5 (15.06)	0.780
^†^Perseverations (%)	0.1 (0.27)	0.2 (0.41)	0.299
HRT (ms)	471.0 (55.29)	516.3 (63.78)	***0.013***
^†^HRTSD	92.3 (27.28)	121.6 (52.99)	***0.010***
^†^Variability	26.4 (17.29)	37.5 (39.78)	0.312
HRT Block Change	0.6 (10.50)	3.2 (8.67)	0.403
HRT Inter Stimulus Interval Change	13.2 (13.30)	14.5 (16.84)	0.773

*MCI, mild cognitive impairment; SCD, subjective cognitive decline; HRT, hit reaction time; HRTSD, hit reaction time standard deviation. Data presented as Mean (Standard deviation). ^†^Comparisons were conducted using the Mann-Whitney *U* Test. Items without labels were conducted by the independent *T*-tests. *P* values < 0.05 are in bold and italicized*.

**Figure 1:**
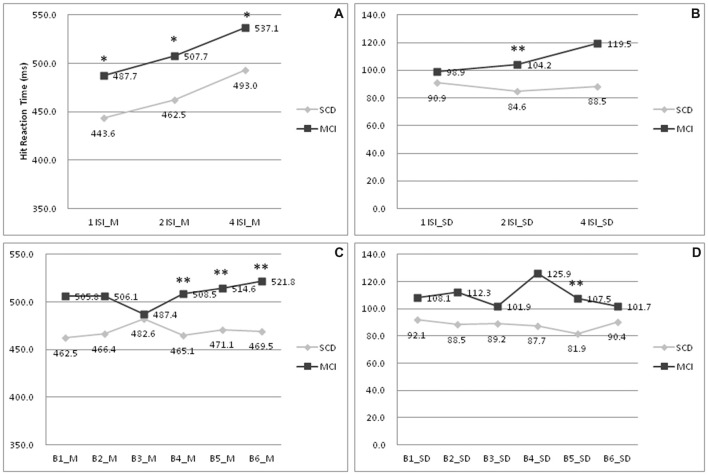
Comparisons of hit reaction time (HRT) profiles between patients with SCD and MCI. **(A)** Mean of HRT under 1/2/4 ISI; **(B)** Standard deviation of HRT under 1/2/4 ISI; **(C)** Mean of HRT under B1-6; **(D)** Standard deviation of HRT under B1–6. MCI, mild cognitive impairment; SCD, subjective cognitive decline; M, mean; SD, standard deviation; 1/2/4 ISI, 1/2/4 s interstimuli interval; B1–6:1st to 6th block. *p < 0.05 on comparisons between subjects with MCI and SCD. **p < 0.01 on comparisons between subjects with MCI and SCD.

To further delineate the profiles of HRT, changes within each ISI and block were analyzed. In Figure 1A, subjects with MCI showed a longer HRT across all ISI (*p* = 0.012–0.036; Cohen’s *d* = 0.68–0.74). In Figure 1B, subjects with MCI showed a wider HRTSD than those with SCD at the 2 s ISI (*p* = 0.009; Cohen’s *d* = 0.73). In SCD subjects, the HRT differed significantly across each ISI (*p* = < 0.001–0.030). In MCI subjects, the HRT differed on comparing values between 1 s ISI to 4 s ISI (*p* = 0.007), 2 s ISI to 4 s ISI (*p* = 0.033), but not 1 s ISI to 2 s ISI (*p* = 0.240). In Figure 1C, subjects with MCI developed a longer HRT than those with SCD at the point of fourth to sixth blocks (*p* = 0.001–0.006; Cohen’s *d* = 0.83–0.99). In Figure 1D, the HRTSD of MCI subjects at the point of fifth block also was larger than that of SCD subjects (*p* = 0.001; Cohen’s *d* = 0.93). There was no interaction between SCD/MCI categorization with variables including ISI, BC, and their standard deviation (*p* = 0.055–0.949).

**Figure 2:**
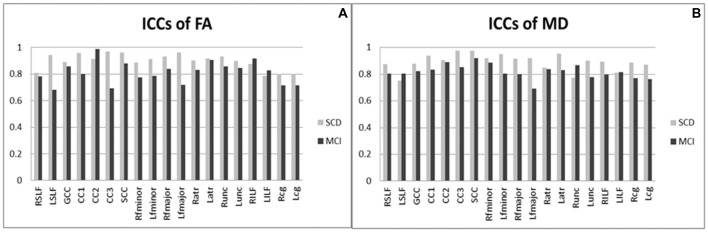
Intra-class correlation coefficients of diffusion tensor imaging results (DTI) among patients with SCD and MCI. **(A)** ICCs of FA; **(B)** ICCs of MD. MCI, mild cognitive impairment; SCD, subjective cognitive decline; ICCs, intra-class correlation coefficients; FA, fractional anisotropy; MD, mean diffusivity; R/L, right/left; SLF, the superior longitudinal fasciculus; GCC, the genu of the corpus callosum; CC1/2/3, the body of the corpus callosum (portion 1/2/3); SCC, the splenium of the corpus callosum; fminor, the forceps minor; fmajor, the forceps major; atr, the anterior thalamic radiations; unc, the uncinate fasciculus; ILF, the inferior longitudinal fasciculus; cg, the cingulum. All ICC were of *P* values < 0.05.

Figure 2 shows ICCs of DTI results, indicating substantial or excellent agreement for diffusion parameters (i.e., FA and MD) on the basis of generally high ICCs (all *p* < 0.05). Overall, the value of each variance follows as the order “Var (β) > Var (ε) > Var (α),” indicating the fact that the majority of variability derived majorly from the differences among the subjects, and a negligible effect from DTI parameters itself. This implies that differences in ICC observed between these two groups are driven by the between-subject variation rather than by the scanning technique being less reliable in one group than the other. For FA in subjects with SCD (Figure 2A), 18 (95%) atlas ROIs had an ICC of 0.8 or above. In subject with MCI, 10 (53%) ROIs had an ICC of 0.8 or above. The rest ROIs were all above 0.6, indicating substantial agreement from test-retest reliability. For MD (Figure 2B), 17 (89%) and 2 (11%) ROIs had ICCs above 0.8 and 0.6–0.8 in the SCD subjects; the corresponding number was 14 (74%) and 5 (26%) in the MCI subjects. In both SCD and MCI subjects, mean ICCs were very similar across metrics (SCD: FA mean ICC = 0.90; MD mean ICC = 0.89; MCI: FA mean ICC = 0.81; MD mean ICC = 0.82).

Table 3 shows comparisons of CPT3 parameters between subjects with SCD and MCI. Subjects with MCI had lower FA values within the left forceps major (*p* = 0.001; Cohen’s *d* = 0.87) and the left uncinate fasciculus (*p* = 0.029), yet higher FA values within the right inferior longitudinal fasciculus (*p* = 0.037) on comparing to those from SCD. There were significantly higher MD values within the bilateral forceps minors in subjects with MCI than those with SCD (*p* = 0.031–0.046). To solve the multiple comparisons problems, an appropriate threshold was selected to be 0.05/19 = 0.0026, leaving the FA values within the left forcep major to be the only region with significant between-group differences.

**Table 3:** Comparisons of diffusion tensor imaging parameters between patients with SCD and MCI.

Regions of interest	SCD (*n* = 39)		MCI (*n* = 15)		*P* value for FA comparison	*P* value for MD comparison
	FA	MD	FA	MD		
Superior longitudinal fasciculus—Rt	0.47 (0.060)	0.77 (0.046)	0.47 (0.066)	0.78 (0.025)	0.878	0.243
—Lt	0.50 (0.073)	0.74 (0.022)	0.50 (0.053)	0.74 (0.036)	0.858	0.808
Corpus callosum —Genu	0.78 (0.058)	0.83 (0.073)	0.78 (0.052)	0.82 (0.064)	0.870	0.798
—Body 1	0.70 (0.074)	0.85 (0.112)	0.71 (0.053)	0.87 (0.124)	0.727	0.562
—Body 2	0.70 (0.051)	0.83 (0.064)	0.66 (0.088)	0.88 (0.110)	0.144	0.107
—Body 3	0.70 (0.064)	0.87 (0.084)	0.72 (0.052)	0.86 (0.057)	0.179	0.726
—Splenium	0.75 (0.102)	0.89 (0.151)	0.71 (0.108)	0.90 (0.157)	0.236	0.761
Forceps minor—Rt	0.44 (0.068)	0.82 (0.057)	0.44 (0.059)	0.86 (0.048)	0.960	***0.031***
—Lt	0.41 (0.064)	0.81 (0.067)	0.39 (0.044)	0.85 (0.053)	0.189	***0.046***
Forceps major—Rt	0.49 (0.115)	0.82 (0.091)	0.46 (0.050)	0.80 (0.063)	0.189	0.566
—Lt	0.52 (0.120)	0.84 (0.127)	0.44 (0.046)	0.81 (0.053)	***0.001*^†^**	0.398
Anterior thalamic radiation—Rt	0.45 (0.061)	0.78 (0.045)	0.47 (0.075)	0.79 (0.067)	0.325	0.920
—Lt	0.46 (0.072)	0.77 (0.075)	0.47 (0.096)	0.78 (0.129)	0.446	0.609
Uncinate fasciculus—Rt	0.36 (0.093)	0.88 (0.053)	0.33 (0.063)	0.89 (0.095)	0.216	0.440
—Lt	0.42 (0.075)	0.83 (0.047)	0.37 (0.071)	0.85 (0.055)	***0.029***	0.179
Inferior longitudinal fasciculus—Rt	0.49 (0.048)	0.95 (0.132)	0.52 (0.046)	0.94 (0.117)	***0.037***	0.857
—Lt	0.55 (0.055)	0.86 (0.075)	0.56 (0.048)	0.86 (0.051)	0.473	0.903
Cingulum—Rt	0.45 (0.052)	0.84 (0.083)	0.45 (0.032)	0.82 (0.040)	0.892	0.465
—Lt	0.43 (0.057)	0.80 (0.056)	0.42 (0.032)	0.80 (0.035)	0.541	0.696

*MCI, mild cognitive impairment; SCD, subjective cognitive decline; FA, fractional anisotropy; MD, mean diffusivity; Rt, right; Lt, left. FA Data presented as Mean (Standard deviation). MD Data presented as Mean (Standard deviation) expressed in units of m^2^s^−1^ × 10^−9^. Comparisons were all conducted by independent T-tests. P values < 0.05 are in bold and italicized. ^†^Significance after the Bonferroni correction*.

Table 4 shows correlation analysis between DTI parameters and HRT/HRTSD. Among significant correlations related to HRT, FA values within the genu of the corpus callosum and left forceps minor had inverse correlations. On controlling age and education, FA value within the left forceps minor remained to be significant (correlation coefficient = −0.378; *p* = 0.006). We didn’t identify significant correlations between MD values and HRT. Among significant correlations related to HRTSD, MD values within the body of corpus callosum had positive correlations. On controlling age and education, MD value within the body of corpus callosum remained to be significant (correlation coefficient = 0.353; *p* = 0.010).

**Table 4:** Correlation analysis between diffusion tensor imaging parameters and HRT performances.

	HRT				HRTSD			
	FA		MD		FA		MD	
Regions of interest	*r*	*P* value	*r*	*P* value	*r*	*P* value	*r*	*P* value
Superior longitudinal fasciculus—Rt	−0.138	0.321	−0.140	0.312	−0.059	0.669	−0.086	0.535
—Lt	−0.046	0.741	−0.214	0.121	−0.038	0.784	−0.068	0.625
Corpus callosum—Genu	−0.279	***0.041***	0.090	0.515	−0.102	0.464	−0.078	0.573
—Body 1	−0.017	0.905	0.093	0.505	0.012	0.931	0.074	0.597
—Body 2	−0.027	0.847	0.200	0.147	−0.263	0.054	0.386	***0.004*^‡^**
—Body 3	0.076	0.583	0.036	0.797	0.060	0.665	−0.055	0.690
—Splenium	−0.028	0.842	0.012	0.934	−0.067	0.632	0.032	0.819
Forceps minor—Rt	−0.259	0.059	0.241	0.079	−0.219	0.111	0.164	0.235
—Lt	−0.378	***0.005*^‡^**	0.073	0.602	−0.104	0.456	−0.058	0.677
Forceps major—Rt	0.220	0.110^‡^	0.045	0.746	0.061	0.664	−0.204	0.139
—Lt	−0.027	0.849	0.026	0.853	−0.191	0.167	−0.122	0.380
Anterior thalamic radiation—Rt	0.239	0.082	−0.112	0.420	0.186	0.179	0.089	0.522
—Lt	−0.038	0.784	−0.005	0.969	0.096	0.489	−0.062	0.657
Uncinate fasciculus—Rt	0.011	0.937	−0.048	0.969	−0.021	0.879	−0.012	0.934
—Lt	−0.034	0.806	0.025	0.860	−0.010	0.942	0.076	0.538
Inferior longitudinal fasciculus—Rt	0.146	0.293	0.179	0.196	0.201	0.145^‡^	−0.062	0.658
—Lt	0.139	0.316	0.111	0.423	0.160	0.249	0.151	0.277
Cingulum—Rt	0.051	0.712	0.177	0.201	0.198	0.151	0.017	0.904
—Lt	−0.135	0.331	0.054	0.699	0.040	0.772	0.011	0.937

*HRT: hit reaction time; HRTSD, hit reaction time standard deviation; FA, fractional anisotropy; MD, mean diffusivity; Rt, right; Lt, left. FA. *P* values <0.05 are in bold and italicized. *r*, Pearson correlation coefficient. ^‡^*P* < 0.05 on the partial correlations on controlling of age and education*.

To further pinpoint the neuronal substrates of HRT in CPT3, data of all SCD/MCI subjects were entered into stepwise linear regression analysis. The independent variables included: (i) FA values within the genu of the corpus callosum; (ii) FA values within the left forceps minor; (iii) demographic data including age, education, gender; and (iv) total scores of CASI.

Regarding HRT, the analysis found that FA value within the left forceps minor (Lfminor_FA; *β* = −0.378, *t* = −2.947, *p* = 0.005) significantly predicted its value (*R*^2^ = 0.143, *F*_(1,52)_ = 8.68, *p* = 0.005). Overall, the model accounted for 14.3% of the variance. The regression model was listed as below.

HRT = 639.2 − 382.1 * Lfminor_FA

*R*^2^ = 0.143, *p* = 0.005

Age, education, gender and total scores of CASI didn’t change the results of those regression models.

**Figure 3:**
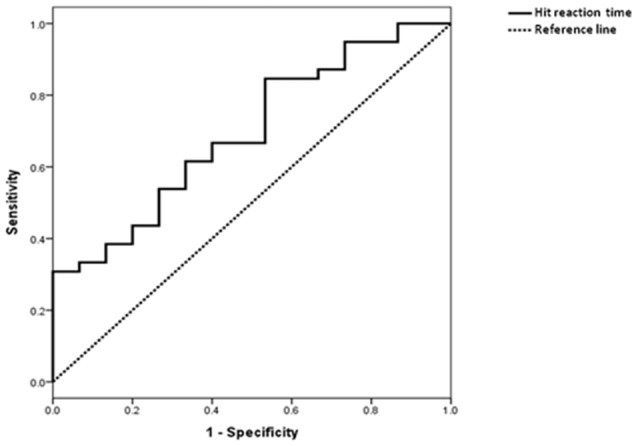
Receiver operating characteristic (ROC) curve of HRT in differentiating subjects with MCI from those with SCD.

ROC curve was shown in Figure 3. The AUC was 0.70 (*p* = 0.028). Based on the maximum Youden index, the optimum cut-off point for HRT was 515.8 ms, with a sensitivity of 85% and specificity of 47%.

## Discussion

Our study identified that subjects with SCD and MCI presented differing visual attention performances in the presence of subtle microstructural changes. The significant correlations between HRT and DTI parameters provide valuable information in delineating sustained attention network. On the basis of no significant group—HRT interaction, the independent role of CPT3 was confirmed, and its application may serve as part of references for designing protocol in identifying subjects who are at risk of future cognitive decline.

The current study identified that MCI subjects had a significantly longer HRT in CPT3 than those with SCD, a result that was distinct and independent from neuropsychological tests primarily applied in standard diagnostic paradigm. Such discernible changes existed in overall comparisons and appeared to be more evident at the point of fourth to sixth blocks, suggesting a phasic change in alertness and defective sustained attention developed around 12 min since the start of test among subjects with MCI. It was attributed mainly onto sustained attention deficits due to the fact that such between-group differences were getting more robust as the CPT3 continued on. In another viewpoint, impaired vigilance may also contribute findings similar to our results. A significant correlation between HRT and HRTSD suggested the possibility that our respondents demonstrated a considerable variability within each individual block. Moreover, those with MCI also expressed a longer HRT across variable ISIs. This indicated MCI subjects would tend to have slower responses regardless varying levels of stimulus frequency. Although we didn’t observe distinct pattern of omission/commission errors, vigilance in MCI subjects was likely to be impaired as they were unable to maintain performances during variable lagging period between signal appearance and internal expectation. We therefore proposed that sustained attention and vigilance were highly intercalated as examined in CPT3. Our current findings provided interesting contrast with a research aiming on longitudinal assessment of neuropsychological tests, in which highly-educated elderly are prone to demonstrated reduced performances in tests with eminent components of executive functions, memory, but not attention (Elkana et al., 2016). Another longitudinal study also stressed that declines in episodic memory and executive function accelerated 7 and 2–3 years before diagnosis of AD, respectively (Grober et al., 2008). While a prevailing body of evidences pointed out impaired memory and/or execution among SCD/MCI subjects (Grober et al., 2008; Sinai et al., 2010; Elkana et al., 2016), some literatures underpinned attention deficits to be one of the non-memory domains to be involved than traditionally conceptualized (Saunders and Summers, 2010, 2011). In a cross-section study, Saunders and Summers (2010) reported that SCD subjects showed better performance than MCI patients in sustained and selective attention. In their subsequent longitudinal study, it was furthermore concluded that the decline in simple sustained attention in amnestic MCI and non-amnestic MCI groups and divided attention in amnestic MCI may be early indicators of dementia conversion (Saunders and Summers, 2011) The aforementioned evidences were in line with our main findings that the complex attention may be one unattended cognitive domain vulnerable to early neurodegenerative process. As sustained attention is defined as the ability to maintain the focus of cognitive activity over time on a given task or stimulation (Sarter et al., 2001), it is expected to highly interact with encoding process of memory implement (Chun and Turk-Browne, 2007). Therefore, attention deficits would be expected along with memory degradation. Our clinical observations also supported this hypothesis, as SCD subjects who complained “memory problems” may frequently report frustration during handling work with more cognitive demands in other non-memory function, such as attention or information processing. Hence, a reliable tool in measuring attention function (e.g., CPT3) would be warranted for subjects with cognitive complaints.

Previous literatures aiming on DTI evaluations among subjects with SCD or MCI are inconsistent. This is highly likely due to variable approaches of neuropsychological tests in addition to their presumed heterogeneous pathologies. In a research where cognitive deficits of SCD subjects were confined within verbal memory tests but with intact global cognition, there were widespread WM alterations on comparing to the normal control (Li et al., 2016). In another two cohorts where cognitive evaluation focused on global cognition and memory function, there was limited or no difference from comprehensive DTI measures (Kiuchi et al., 2014; Wang X. N. et al., 2016). Moreover, some other study depicted an intermediate pattern of DTI parameter modification between normal controls and MCI, in whom their cognitive profiles were diagnosed by comprehensive neuropsychological tests (Hong et al., 2015). Each subject in our current study had received a neuropsychological test aiming on major cognitive domains, therefore stringently confining their cognitive performance and relevant cerebral degenerative process within a very early stage. Compared with SCD subjects, those with MCI had a lower FA value within the left forceps major and a trend of lower FA values within the left uncinate fasciculus but higher FA values within the right inferior longitudinal fasciculus. Subtle changes within these tracts might indicate coexistence of both Wallerian degeneration and retrogenesis progression. While a lowered FA value within the left forceps major and uncinate fasciculus appeared to be consistent with previous report (Stenset et al., 2011) a paradoxical elevation of FA value within the right inferior longitudinal fasciculus could be the consequence related to a micro-inflammation state and/or their crossing fiber property. FA value elevation during cerebral degenerative process was proposed as compensatory glial activation in response to inflammatory effects of amyloid deposition (Racine et al., 2014). Such hypothesis was also supported by the models of brain abscess (Nath et al., 2007). Another possible explanation was hypothesized that selective degeneration of short interneurons but spared long projection neurons might happen alongside crossing fibers; a loss of collateral projections would therefore contribute FA value elevation (Racine et al., 2014).

Another interesting main finding is that FA values appeared to be more sensitive than MD values by between-group comparisons of DTI parameters. Theoretically, FA measures the overall directionality of water diffusion and reflects the axonal membrane and myelin integrity, whereas MD represents barriers to free water diffusion such as axon damages during neurodegenerative process (Alves et al., 2015). Previous studies indicated that MD and FA differences usually coexisted by comparing DTI parameter of subjects with SCD and MCI (Kiuchi et al., 2014; Hong et al., 2015). Some study also indicated a prevailing role of MD than FA regarding its greater spatial involvement and clinical relevancy (Kiuchi et al., 2014). However, some other researchers highlighted FA value to be a unique biological predictor for memory performances among subjects with SCD and MCI (Grambaite et al., 2010). A wider distribution of FA modifications than MD was also reported (Wang et al., 2012). In addition, some other studies also addressed that FA was a more sensitive measure than other diffusion parameters in differentiating patterns of white matter changes (Tu et al., 2017) and the subtype of MCI (Haller et al., 2013). Regarding clinical relevancy, our study supported that both FA and MD values can serve as useful biological determinants in terms of cognitive performances, as those relevant microstructural integrity of the genu/body of the corpus callosum and left forceps minor correlated with HRT or HRTSD. The results highlighted these neuronal substrates related with implement of sustained attention among SCD/MCI subjects, and were consistent to current opinion related to alerting network. In one line of evidences, experimental drug studies with humans and monkeys showed that norepinephrine governed alerting network (Morrison and Foote, 1986; Melasch et al., 2016). The cortical norepineprhine pathway includes the anterior insula/frontal operculum, medial orbitofrontal cortex (Melasch et al., 2016). In another way, several important regions including thalamus, anterior cingulate, and supplementary motor cortex were proven to be modulated by the contingent negative variation, an important negative potential observed by electroencephalography during response anticipation (Nagai et al., 2004).

The result of correlation analysis also indicated that subjects’ reaction time might be modulated by later attentional processes more than that primarily reflected in reaction time. Our study showed an important observation that there were remarkable correlations between HRT and anterior interhemispheric fibers. As HRT performances of MCI subjects slowed during later blocks of CPT3, it is reasonable to infer that the expectancy of stimulus emergence may be modified by the executive network, in which the anterior cingulate cortex and associated projecting fibers governs the heading of target detection (Petersen and Posner, 2012; Neta et al., 2014). Consistently, previous literatures suggested the association between CPT performances and FA modifications within several regions, including corpus callosum, cingulum, frontostriatal tracts, and superior longitudinal fasciculus (Segura et al., 2010; Takahashi et al., 2010; Chiang et al., 2015). In another aspect of considerations, a slower HRT across all ISIs may reflect impaired alerting network. While reaction time, deemed to be the major component of alerting network, was once regarded to be heavily associated with the right hemisphere, a growing body of evidences supported the lateralization effect may vary in terms of the warning signal effects and/or temporal expectancies (Fan et al., 2005). Interestingly, our report suggested that microstructural integrity within the left forceps minor best predicted the performances of HRT. As subjects who underwent CPT3 are unable to predict the timing of target appearance, the results were consistent with the findings reported by Coull et al. (2000) where activation of the left frontal and bilateral orbitofrontal cortex was noted during the condition of breached temporal expectancy. Several lines of experiments may provide additional information regarding lateralization effect (Parasuraman et al., 1998; Fan et al., 2005; Petersen and Posner, 2012). The tonic alertness, a relatively sustained activation level with a slower effect, often is associated with right lateralization process (Parasuraman et al., 1998). The phasic alertness, a short-lived activation with higher temporal frequencies, is more likely to be associated with left hemisphere implement (Fan et al., 2005; Petersen and Posner, 2012). Taken together, neuronal substrates related to HRT in CPT3 suggested the involvement of frontal-predominant processes with a left lateralization effect that may be attributed by the unpredicted temporal expectancy property of the task.

Strength of the current study included the study design, which compared and incorporated findings from comprehensive neuropsychological tests and neuroimaging assessment. The results were derived from a cohort in which subjects were properly categorized through stringent standard neuropsychological tests. Subjects in our cohort were free from remarkable white matter hyperintensities, making the possibility of vascular cognitive impairment less likely. The majority of our MCI cases were categorized as amnestic type, indicating our observation would fit into current concept of pathology related to AD rather than frontotemporal lobar degeneration. Therefore, homogeneity of cognitive status was expected to be purified by current diagnostic repertoire. However, several limitations should be considered in our research work. In subject recruitment, this study was limited by a lack of a control group; as such, the independent influence of “spontaneous report of cognitive deficits” cannot be ascertained. The subject number was limited, therefore raising concern that whether the results might be biased by the small sample size or the compensatory cognitive function as expected to happen during the early stage of SCD/MCI. The heterogeneity of underlying pathology remained to exist, as the speed of cognitive decline and genetic associations may vary according to age-of-onset (Jessen et al., 2014a). It is therefore incorporating biomarkers related to AD should be considered in the future studies. However, we are convinced that our report to be of clinical significance due to the fact that the majority of SCD subjects showed cognitive concern and most MCI subjects were identified to be amnestic type, which have been regarded typical for AD related pathology (McKhann et al., 2011). We were also aware that our current results were derived from a cross-sectional study; a longitudinal follow-up study would be necessary to re-examine the value of CPT3 in predicting underlying neurodegenerative pathology. In neuropsychological aspects, there might be considerations that CPT3 performances were affected by memory retrieval deficits in most of our MCI cases. We therefore clarified group—HRT interaction to be insignificant and confirmed the independent role of CPT3. Finally, as CPT3 may not fully clarify phenotypes associated with other complex attention (e.g., selective or divided attention), nor can it explore attention process in terms of individual network (e.g., alerting, executive and orienting networks), deployment of other attention measurement, such as the Attention Network Test (Fan et al., 2002), may be considered in the future study.

## Conclusion

Our report suggested the independent role of the CPT3, which showed a discernible visual attention performance between subjects with SCD and MCI. The MCI subjects had a longer HRT across all ISIs more profoundly in later BC, corroborating that impaired vigilance and sustained attention are early cognitive markers in differentiating MCI from SCD. FA measures appeared to be a DTI parameter more sensitive than MD values in detecting microstructural changes, as significant FA modifications within the left forceps major was observed. The neuronal substrates related with HRT highlighted the role of anterior interhemispheric fibers in sustained attention implementation, and the left lateralization effect reflected unpredicted temporal expectancy property in CPT3.

## Author Contributions

M-CT and Y-HH: study concept and design, analysis, interpretation and drafting the manuscript. W-HH, JFD and Y-HH: neuropsychological test assessment and interpretation. C-PL and C-FH: study concept and critical review. All authors contributed in writing the manuscript.

## Conflict of Interest Statement

The authors declare that the research was conducted in the absence of any commercial or financial relationships that could be construed as a potential conflict of interest.
